# Diverse Novel Viruses Coinfecting the Tropical Ornamental Plant *Polyscias balfouriana* in China

**DOI:** 10.3390/v14061120

**Published:** 2022-05-24

**Authors:** Yuxin Ma, Haiyan Che, Shengfeng Gao, Yating Lin, Shifang Li

**Affiliations:** 1 Environment and Plant Protection Institute, Chinese Academy of Tropical Agricultural Sciences, Haikou 571101, China; yuxin.ma@catas.cn (Y.M.); chehaiyan2012@126.com (H.C.); lytyazai@126.com (Y.L.); 2 State Key Laboratory Breeding Base of Green Pesticide and Agricultural Bioengineering/Key Laboratory of Ministry of Education, Guizhou University, Guiyang 550025, China; 3 Spice and Beverage Research Institute, Chinese Academy of Tropical Agricultural Sciences, Wanning 571533, China; gsfkl@163.com; 4 State Key Laboratory for Biology of Plant Diseases and Insect Pests, Institute of Plant Protection, Chinese Academy of Agricultural Sciences, Beijing 100193, China

**Keywords:** *Polyscias balfouriana*, virus discovery, coinfection, ornamental plant, *Betaflexiviridae*, *Closteroviridae*, *Capillovirus*, *Citrivirus*, *Crinivirus*

## Abstract

The viromic profile of *Polyscias balfouriana* cv. Marginata, a perennial woody and ornamental plant, was determined using ribosomal RNA-depleted total RNA (rRNA-depleted totRNA) sequencing. Five viruses (i.e., polyscias mosaic virus, PoMV; one potential novel rhabdovirus; and three novel viruses of *Betaflexiviridae* and *Closteroviridae*) were detected and prevalence-surveyed in Hainan province, China. The genomes of polyscias capillovirus 1 (PCaV-1) and polyscias citrivirus 1 (PCiV-1) of family *Betaflexiviridae* were completed, and the genomes of polyscias crinivirus 1 (PCrV-1) of *Closteroviridae* were nearly completed lacking the 5′ and 3′ termini. PCaV-1 shares 68% genome nucleotide (nt) identity and 66% replicase (Rep) amino acid (aa) identity with homologues in apple stem grooving virus (ASGV). PCiV-1 shares 65% genome nt identity and 64% Rep aa identity with homologs in citrus leaf blotch virus (CLBV). Meeting the species demarcation criteria, PCaV-1 and PCiV-1 were considered to be new species in genera *Capillovirus* and *Citrivirus*, respectively. PCrV-1 shares high genome nt identity (62%), heat shock protein 70-like protein (HSP70h) and RNA-dependent RNA polymerase (RdRp) aa identity (78–80%) with homologues in tomato chlorosis virus (ToCV). We tentatively consider PCrV-1 to be an unclassified member of the *Crinivirus* genus. PoMV, PCaV-1, PCiV-1, and PCrV-1 are the prevalent viruses with >73% occurrence in the Xinglong Tropical Botanical Garden, Hainan, China.

## 1. Introduction

High-throughput sequencing (HTS) for viral discovery has been successfully applied to various plants, including economically important crops, perennial fruit trees, vegetables, and, more recently, wild plants [1,2,3,4,5,6,7]. However, viromic analysis has not been extensively applied to tropical ornamental plants.

**Figure 1:**
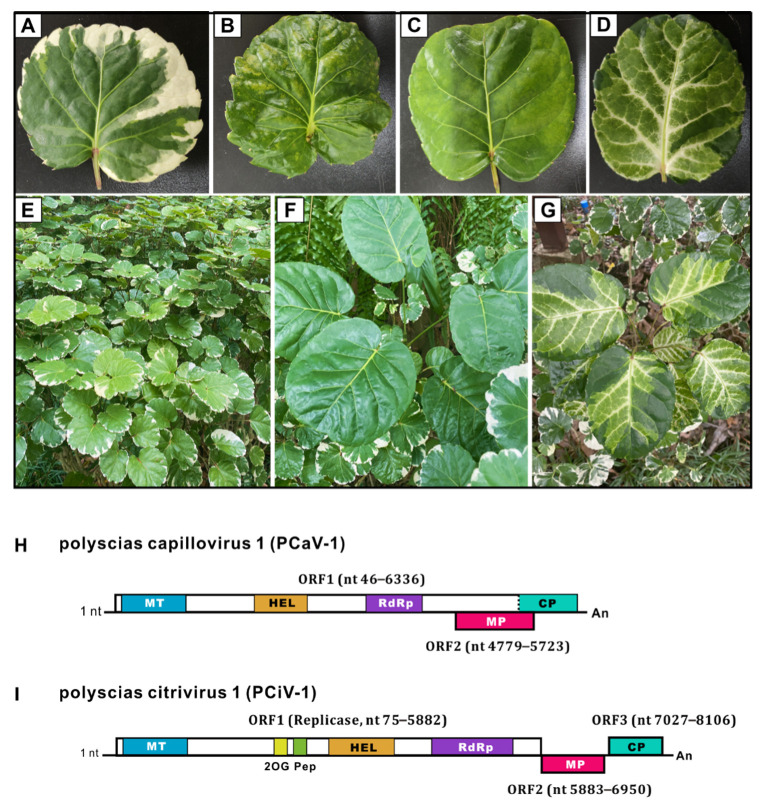

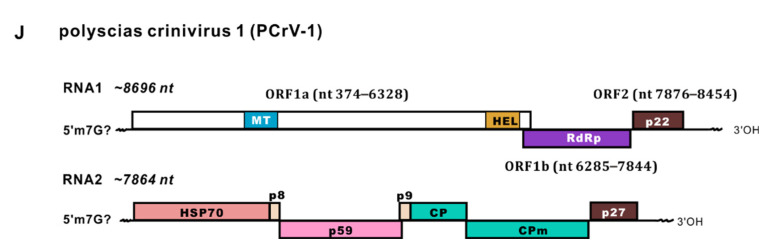
(**A**–**G**) *Polyscias balfouriana* samples of different cultivars with different morphologies collected from Xinglong Tropical Botanical Garden, Hainan Province, China; (**H**–**J**) schematic representation of the genomic organisation of three newly identified RNA viruses. Most of the samples collected in this study with rounded leaves edged in white are shown in (**A**,**E**), which are *P. balfouriana* cv. Marginata; a few samples were (**C**,**F**) *P. balfouriana* and (**D**,**G**) *P. balfouriana* cv. Pennockii; the leaflets have an attractive blend of ivory, yellow, and dark green patches. Predicted open reading frames (ORFs) are represented by boxes, with nucleotide (nt) coordinates indicated. Conserved motifs, domains, and viral proteins are indicated within ORFs by different colours (**H**–**J**). Viral methyltransferase (MT, pfam 01660, cl03298), viral helicase (HEL, pfam 01443), and RNA-dependent RNA polymerase_2 (RdRp, pfam 00978), 2OG-FeII Oxy superfamily (2OG, cl21496), peptidase C23 superfamily (Pep, cl05111), coat protein (CP), minor CP (CPm), movement protein (MP), and heat shock protein 70-like protein (HSP70h). Predicted proteins with unknown functions are also indicated, including a 22 kDa protein in RNA1 of PCrV-1, and an 8 kDa protein (p8, nt 2044–2259), a 60 kDa protein (p59, nt 2199–3752, 517 aa), a 9 kDa protein (p9, nt 3734–3973), and a 27 kDa protein (p27, nt 6761–7459, 232 aa) in RNA2; 5′ and 3′ noncoding regions are shown by black lanes at both extremities. An, poly(A) tail.

*Polyscias balfouriana*, also known as *Aralia balfouriana* or balfour aralia, is a perennial woody plant usually cultivated as an ornamental species in gardens or indoors. This plant is attractive and highly valued for its beautiful foliage. The main cultivar is *P. balfouriana* Marginata, imported in 2001 from untraceable origin, and now with a small number of plantings in Hainan, China. So far, viral studies of the *P. balfouriana* are scarce, with only one badnavirus (polyscias mosaic virus, PoMV) being reported from the *Polyscias fruticosa* L. (Ming aralia) [8]. In a viromic study of diverse tropical plants in China in 2019, a *P. balfouriana* Marginata sample (Figure 1A) was collected from Xinglong Tropical Botanical Garden (18°44′06.6″ N 110°11′36.1″ E), Hainan province, China. Double-stranded RNA (dsRNA) was purified from the sample that pooled *P. balfouriana* Marginata and other plants and sequenced on an Illumina platform with a paired-end 150 bp read length [7]. We obtained a total of 103,474 clean reads assembled into 132 contigs, 45 of which were viral contigs by BLASTx analysis. Thirteen contigs with lengths ranging from 250 to 604 nucleotides (nt) hit the viruses of the *Capillovirus* and *Citrivirus* genera in the *Betaflexiviridae* family by sharing 54.6% to 92.6% amino acid (aa) identity with different viral proteins. Similarly, 32 contigs with lengths ranging from 288 to 1343 nt hit the tomato chlorosis virus (ToCV) of the *Crinivirus* genus in the *Closteroviridae* family by sharing 32.1% to 85.9% aa identity. We thus preliminarily predicted the presence of multiple new viruses from the *Betaflexiviridae* and *Closteroviridae* families in the balfour aralia sample.

On the basis of dsRNA sequencing results, to further determine the virome of *P. balfouriana*, we conducted rRNA-depleted totRNA sequencing to identify novel viruses, molecularly characterise them, and assess their occurrence in Hainan province, China.

## 2. Materials and Methods

### 2.1. Plant Materials and HTS

The original *P. balfouriana* sample showing no viral symptoms compared to the healthy plants was collected from Xinglong Tropical Botanical Garden, Hainan province, China, in 2019 (Figure 1A). dsRNA was purified from 0.75 g of fresh leaf material and sequenced (2 × 150 bp) on an Illumina Novaseq 6000 instrument for the preliminary screening of viruses using previously described methods. In 2021, we conducted rRNA-depleted totRNA sequencing on the same individual *P. balfouriana* plant for detailed viromic screening. Total RNA extraction, rRNA depletion, and subsequent library preparation were conducted at LC-Bio Technology Co., Ltd., Hangzhou, China using previously described methods [9]. Briefly, 2 μg of total RNA was treated for the removal of rRNA using the Epicentre Ribo-Zero Gold Kit (Illumina, San Diego, CA, USA). The rRNA-depleted RNAs were then fragmented using the Magnesium RNA Fragmentation Module (New England Biolabs, Ipswich, MA, USA) at 94 °C for 5 to 7 min. The cleaved RNA fragments were reverse-transcribed into cDNA using SuperScript™ II Reverse Transcriptase with random hexamer primer (Invitrogen, Carlsbad, CA, USA). PCR products were sequenced (2 × 150 bp) on an Illumina Novaseq 6000 instrument following the manufacturer’s procedure [9].

### 2.2. Data Processing and Virus Annotation

After the adapter trimming and filtering of raw reads, clean reads obtained from dsRNA sequencing were de novo assembled into contigs using IDBA-UD assembler v1.1 [10]. For rRNA depleted totRNA sequencing, since the reference genome of *Polyscias balfouriana* is not currently available, the genomic sequence of *Panax japonicus* from the same family *Araliaceae* (https://ftp.ncbi.nlm.nih.gov/genomes/all/GCA/020/205/505/GCA_020205505.1_ASM2020550v1/, accessed on 4 October 2021), was used for host-derived read elimination using HISAT v2.1.0 [11] to reduce the size of the dataset. The remaining reads were de novo assembled into contigs using Trinity v2.13.3 [12], and annotated using BLASTn and BLASTx [13] against the nonredundant nucleotide (nt) and protein (nr) GenBank databases [14] with a conservative e-value cut-off of 10^−4^.

### 2.3. Completion of Genomes of Viruses Discovered from HTS

The genomes of the newly identified viruses were reconstructed by reverse-transcription polymerase chain reaction (RT-PCR) with several pairs of specific primes designed on the basis of the generated viral contigs (Table S1) using TransScript^®^ One-Step gDNA Removal and cDNA Synthesis SuperMix (Transgen, Beijing, China) and SuperStar Max DNA Polymerase (GenStar, Beijing, China). The full genomes of the two new viruses of *Betaflexiviridae* were obtained with 5′ and 3′ untranslated regions (UTRs) completed, whereas the genome of the novel crinivirus of *Closteroviridae* lacked the 5′ and 3′ termini. The LD-oligo(dT) primer (5′-TGTGTTGGGTGTGTTTGGTTTTTTTTTTTTTTT-3′) was used to synthesise the cDNAs of RNA viruses with poly(A) tails, and the dt-rev primer (5′-TGTGTTGGGTGTGTTTGG-3′) paired with gene-specific primers was used to complete the 3′-terminal sequences. The 5′-terminal sequences were amplified using a SMARTer RACE 5′/3′ Kit (Takara Bio, Mountain View, CA, USA; Table S1). The amplified PCR products were either directly sequenced or cloned into the pGEM-T Easy vector (Promega, Madison, WI, USA) before sequencing. The near-complete genomes of the crinivirus were determined on the basis of assemble scaffolds from rRNA-depleted totRNA sequencing.

### 2.4. Genomic Organisation and Phylogenetic Analyses

Open reading frames (ORFs) were predicted using the ORFfinder web tool (https://www.ncbi.nlm.nih.gov/orffinder/ accessed on 4 October 2021), and conserved domains and motifs were identified by searching the Conserved Domain Database (CDD, https://www.ncbi.nlm.nih.gov/Structure/cdd/wrpsb.cgi accessed on 4 October 2021). Pairwise comparisons were performed on the basis of CLUSTALW multiple sequence alignments, and neighbour-joining (NJ) phylogenetic trees were constructed using MEGAX v10.1.8 (Kumar, Stecher, Li, Knyaz, and Tamura, Philadelphia, PA, USA) [15] with 1000 bootstrap iterations.

### 2.5. Analysis of the Occurrence of the Three Novel Viruses

To further investigate the occurrence of the three newly identified viruses, we collected 22 *P. balfouriana* samples (three different cultivars) mainly exhibiting four different leaf morphologies (Figure 1A–G; detailed pictures of each leaf are shown in Figure S2) from Xinglong Tropical Botanical Garden (18°44′06.6″ N 110°11′36.1″ E), Hainan province, China in 2021. RT-PCR was conducted to test the occurrence of five discovered viruses from HTS, with detection primers listed in Table S1.

## 3. Results

### 3.1. Results of rRNA-Depleted totRNA Sequencing and Discovery of Viruses

The rRNA-depleted totRNA sequencing experiment yielded 76,048,918 raw reads (72,691,030 clean reads) with Q20 and Q30 values greater than 93%, and 30,349,892 (41.8%) reads mapped to the host reference genome, while 42,341,138 (58.2%) unmapped reads were de novo assembled into 286,133 contigs. Similar to the preliminary dsRNA sequencing results, BLASTn and BLASTx analyses revealed that 1738 contigs corresponded to viruses belonging to the *Betaflexiviridae* (*Capillovirus* and *Citrivirus* genera), *Closteroviridae* (*Crinivirus* genus), and *Caulimoviridae* and *Rhabdoviridae* (Table 1) families. Of the 1738 contigs, 113 were long (1000–6219 nt) and had good coverage of the viral genomes. Three long contigs of 6219, 5344, and 3704 nt shared ~90% nt identity (95% coverage) with the complete genome of the polyscias mosaic virus (PoMV, NC_055562), a recently identified badnavirus from *Polyscias fruticosa* L. (Ming aralia) [8]. Considering the 7.14% nt divergence of RT + RNAse H sequences, lower than the species demarcation criteria of 20% in the *Badnavirus* genus, the badnavirus found in this study was considered to be PoMV, which was for the first time discovered from *P. balfouriana* in China. One 5855 nt contig shared 60.57% aa identity (96% coverage) with the RdRp of cabbage cytorhabdovirus 1 (YP_010086794), which indicated the presence of a potential novel cytorhabdovirus in the *Rhabdoviridae* family. No further characterisation was conducted for the viruses of *Caulimoviridae* and *Rhabdoviridae*. Thirteen long contigs (1000 to 4873 nt) corresponded to criniviruses with good coverage, and were used to assemble the near-complete scaffolds of polyscias crinivirus RNA1 and RNA2 genomes in this study.

### 3.2. Characterisation of Two Novel Viruses in the Family Betaflexiviridae

The new capillovirus, tentatively given the name polyscias capillovirus 1 (PCaV-1, accession no. ON240063), consists of a positive-sense, single-stranded RNA (+ssRNA) genome of 6447 nt excluding the poly(A) tail with 5′ and 3′ UTRs completed (Figure 1H). Its complete genomic nt sequence shares 68% nt similarity with that of apple stem grooving virus (ASGV) isolate Kiyomi (LC184611) and of citrus tatter leaf virus (CTLV) isolate TL102 (MH108977). The 5′-UTR with a length of 45 nt shares 91% nt identity with that of CTLV (MH108977), and the 3′-UTR with a length of 111 nt shares 74% nt identity with that of breadfruit capillovirus 1 (MW328738). Similar to other capilloviruses, the PCaV-1 genome possesses two ORFs, with ORF1 (nt 46–6336) encoding a 2096 aa polyprotein containing a replication-associated protein (Rep, nt 46–5577, 1856 aa, 214.4 kDa) and a coat protein (CP, nt 5578–6336, 237 aa, 27.4 kDa). Rep and CP proteins share the highest aa identity with homologues of ASGV (AXP83296, 66% for Rep and 80% for CP; Table S2). Three viral conserved domains were found within the ORF1 protein: a viral methyltransferase domain (MT, pfam01660, aa 43–344), a viral RNA helicase domain (HEL, pfam01443, aa 776–1029), and an RNA-dependent RNA polymerase 2 domain (RdRp_2, pfam00978, aa 1244–1488). ORF2 (nt 4779–5732) is nested in ORF1 and encodes a putative movement protein (MP, 317 aa; Figure 1H).

The novel citrivirus, provisionally named polyscias citrivirus 1 (PCiV-1, accession no. ON240064), has an 8306 nt +ssRNA genome excluding the poly(A) tail, with a 74 nt 5′ UTR and a 201 nt 3′UTR (Figure 1I). The whole genome shares 65% nt identity with that of citrus leaf blotch virus isolate HBYD (CLBV, MG572236). Similar to other citriviruses, the genome of PCiV-1 contains three putative ORFs (Figure 1I). ORF1 (nt 75–5882) encodes a putative Rep that contains five conserved domains, namely, MT (pfam01660, aa 44–342), the 2OG-FeII Oxy superfamily (cl21496, aa 841–939), the peptidase C23 superfamily (cl05111, aa 957–1043), the helicase 1 superfamily (cl26263, aa 1134–1348), and the RdRP_2 superfamily (cl03049, aa 1553–1843). ORF2 (nt 5883–6950) and ORF3 (nt 7027–8106) encode potential MP and CP proteins, respectively. Between ORF2 and ORF3, there is a 77 nt intercalated noncoding region. PCiV-1 shares 64% and 72% aa identities with Rep and CP of CLBV (NP 624333 for Rep and NP_624335 for CP, Table S2).

**Figure 2:**
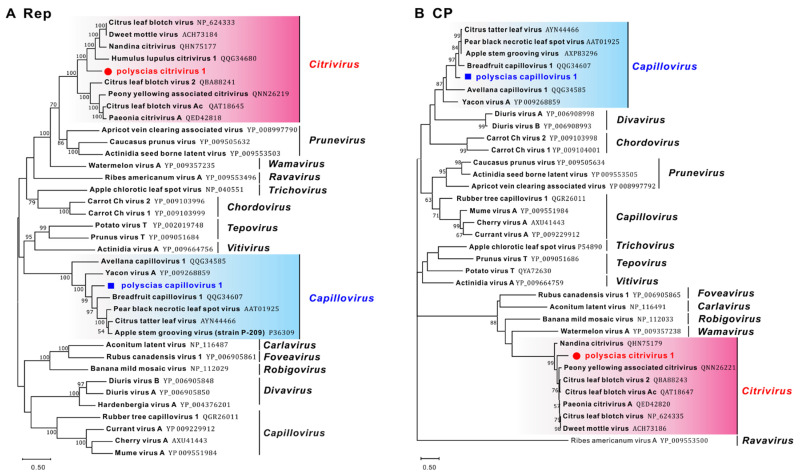
Phylogenetic trees constructed on the basis of (**A**) Rep and (**B**) CP amino acid (aa) sequences of representative members of the *Betaflexiviridae* family. The trees were constructed using the neighbour-joining method, and the statistical significance of branches was evaluated by bootstrap analysis (1000 replicates). Bootstrap values above 50% are shown. Scale bar represents 50% aa sequence divergence. Newly discovered viruses are indicated by red circles (polyscias citrivirus 1, PCrV-1) and blue squares (polyscias capillovirus 1, PCaV-1). Genera are indicated on the right. *Capillovirus* genus (containing PCaV-1), pink background; *Citrivirus*, blue background.

Phylogenetic trees based on the Rep and CP aa sequences of representative members of the *Betaflexiviridae* family showed that these capilloviruses were separated into two clades, and PCaV-1 clustered with ASGV separate from MuVA, CVA, and CuVA (Figure 2), while PCiV-1 clustered with other citriviruses in a single clade.

On the basis of the accepted species demarcation molecular criteria for the family *Betaflexiviridae*, which are of 72% nt identity (or 80% aa identity) for Rep and CP genes [16], polyscias capillovirus 1 and polyscias citrivirus 1 were tentatively considered to be novel species in the *Capillovirus* and *Citrivirus* genera, respectively. Recently, several tentative novel capilloviruses have been identified from perennial woody plants, including loquat [17], birch [18] and rubber [19]. Additionally, some new citriviruses were identified from *Nandina domestica* [20] and ornamental peony [21]. Most capilloviruses and citriviruses have been originally isolated from woody plants, in this case, *P. balfouriana*.

### 3.3. Characterisation of a Novel Virus in the Family Closteroviridae

Another potential novel crinivirus in the *Closteroviridae* family, provisionally named polyscias crinivirus 1 (PCrV-1), was partially molecularly characterised on the basis of long scaffolds assembled from rRNA-depleted totRNA sequencing lacking the 5′- and 3′-terminal sequences. Similar to other criniviruses, PCrV-1 has bipartite genomes of RNA1 (~8696 nt, accession no. ON240065) and RNA2 (~7864 nt, accession no. ON240066) with undefined 5′- and 3′-terminal sequences (Figure 1J). The RNA1 genomic sequence shares 62% nt identity with ToCV isolate TN11 (MF795556), and the RNA2 genome shares 61% nt identity with ToCV-BJ (KC887999). The RNA1 genome has three typical predicted ORFs encoding a 1984 aa polyprotein (ORF1a, nt 374–6328), an RdRp (nt 6285–7844, 520 aa) expressed from ORF1a by a +1 frame shift, and a p22 protein with unknown function (nt 7876–8454, 192 aa). The RNA2 genome consists of seven predicted ORFs, namely, a 62 kDa heat-shock protein 70-like protein (HSP70h, nt 370–2034, 554 aa), an 8 kDa protein (p8, nt 2044–2259), a 60 kDa protein (p59, nt 2199–3752, 517 aa), a 9 kDa protein (p9, nt 3734–3973), a 29 kDa major CP (nt 3970–4743, 257 aa), a 76 kDa minor CP (CPm, nt 4737–6758, 673 aa,) and a 27 kDa unknown protein (p27, nt 6761–7459, 232 aa; Figure 1J).

A close molecular relationship was found among PCrV-1, ToCV, and SSVV on the basis of the identities of encoded proteins (Table S3) and phylogenetic analyses (Figure S1). The accepted species demarcation molecular criteria for the *Closteroviridae* family are 75% aa identity for HSP70h, RdRp, and CP genes. The HSP70h and RdRp genes of PCrV-1 share higher aa identities (78% and 79%) with those of ToCV, while only the CP gene shares 71% aa identity satisfying the criteria.

To further clarify the taxonomy of PCrV-1, we compared the aa identities of HSP70h and Rep between the 13 reported formal species in the genus *Crinivirus* (Table S4). We discovered higher HSP70h and Rep identities (>75%, the criteria) among four viruses: bean yellow disorder virus (BYDV), lettuce chlorosis virus (LCV), tetterwort vein chlorosis virus (TwVCV), and cucurbit yellow stunting disorder virus (CYSDV), and between strawberry pallidosis-associated virus (SPaV) and diodia vein chlorosis virus (DVCV; Table S4). Thus, with no biological data for vector species or serological specificity, we could not determine PCrV-1 to be a new species in the *Crinivirus* genus only on the basis of the sequence identities in this study, although its host is a different perennial woody plant from the herbaceous hosts of ToCV [22]. Therefore, we tentatively considered polyscias crinivirus 1 to be an unclassified member of the *Crinivirus* genus. Since the hosts of criniviruses are generally herbaceous plants [23], *P. balfouriana* is the second known woody host for criniviruses alongside mulberry [24].

### 3.4. Occurrence of Five Discovered Viruses in P. balfouriana in Hainan Province, China

The most prevalent virus in *P. balfouriana* in Xinglong Tropical Botanical Garden in Hainan province is badnavirus PoMV with occurrence of 86%, followed by PCrV-1 (18/22, 82%), PCaV-1 and PCiV-1 (16/22, 73%), and the uncharacterised novel rhabdovirus (32%, 7/22). Only two samples had no target viruses (Figure S2). Although various morphological samples were tested, coinfection was still common, with 14 out of 22 (63.6%) samples being positive for the four target viruses excluding the novel rhabdovirus. Inspired by the phenomenon of the tulip breaking virus being one of five plant viruses causing the colour breaking of tulip flowers, it would be interesting to explore whether the coinfected viruses identified in this study contributed to the appearance of balfour aralia. However, no significant correlations could be concluded between the morphologies and symptoms, and the identified viruses on the basis of the limited data in this study. The high prevalence of the four popular viruses (PoMV, PCaV-1, PCiV-1, PCrV-1) could be due to vegetation propagation from mother plants. Some undiscovered viruses present in the samples may also contribute to the appearances various leaves. Further investigations are necessary to assess the virome, and evaluate the distribution, prevalence, and potential pathogenicity of these new viruses.

## Figures and Tables

**Table 1:** Statistics for rRNA-depleted totRNA sequencing output including the number, length range, and identities of viral contigs annotated by BLASTx analysis.

Family	Genus	Virus	No. of Contigs	Length Range (nt)	Identity Range (%)
*Betaflexiviridae*	*Capillovirus*	Apple stem grooving virus (ASGV)	67	211–2898	46–92
	*Capillovirus*	Citrus tatter leaf virus (CTLV)	15	224–683	26–81
	*Capillovirus*	Yacon virus A (YVA)	24	204–1389	40–83
	*Citrivirus*	Citrus leaf blotch virus (CLBV)	505	201–1027	30–98
	*Citrivirus*	Nandina citrivirus	186	202–1021	40–97
	*Citrivirus*	Citrivirus sp.	20	207–724	29–65
*Closteroviridae*	*Crinivirus*	Sedum sarmentosum crinivirus (SSCV)	74	205–4873	33–88
	*Crinivirus*	Tomato chlorosis virus (ToCV)	48	203–3105	37–89
*Caulimoviridae*	*Badnavirus* *Caulimovirus*	Multiple generic viruses	483	204–6219	22–100
*Rhabdoviridae*	*Nucleorhabdovirus* *Cytorhabdovirus*	Multiple generic viruses	316	201–5855	31–99

## Data Availability

The data presented in this study are available on request from the corresponding author.

## Supplementary Materials

The following supporting information can be downloaded at https://www.mdpi.com/article/10.3390/v14061120/s1: Table S1: primers used for the completion of genomes of PCaV-1 and PCiV-1, and for the detection of the three viruses; Table S2: amino acid identities of Rep and CP between PCaV-1 and PCiV-1, and other members in genera *Capillovirus* and *Citrivirus*; Table S3: amino acid identities of HSP70h, RdRp, and CP between PCrV-1, and other formal and unclassified members in the genus *Crinivirus*; Table S4: amino acid identities of HSP70h (lower left) and RdRp (upper right) between formal species in genus *Crinivirus*; Figure S1: phylogenetic trees constructed on the basis of HSP70h and CP amino acid sequences of formal and tentative members in the *Crinivirus* genus; Figure S2: leaf morphology of 22 *Polyscias balfouriana* samples collected from Xinglong Tropical Botanical Garden, Hainan Province, China in 2021, and the occurrence of the new viruses (PCaV-1, PCiV-1, PCrV-1, PoMV, and the rhabdovirus) discovered in these samples.

## References

[1] Maree H.J., Fox A., Al Rwahnih M., Boonham N., Candresse T. (2018). Application of HTS for Routine Plant Virus Diagnostics: State of the Art and Challenges. Front. Plant Sci..

[2] Villamor D.E.V., Ho T., Al Rwahnih M., Martin R.R., Tzanetakis I.E. (2019). High Throughput Sequencing For Plant Virus Detection and Discovery. Phytopathology.

[3] Maliogka V.I., Minafra A., Saldarelli P., Ruiz-García A.B., Glasa M., Katis N., Olmos A. (2018). Recent Advances on Detection and Characterization of Fruit Tree Viruses Using High-Throughput Sequencing Technologies. Viruses.

[4] Maclot F., Candresse T., Filloux D., Malmstrom C.M., Roumagnac P., van der Vlugt R., Massart S. (2020). Illuminating an Ecological Blackbox: Using High Throughput Sequencing to Characterize the Plant Virome Across Scales. Front. Microbiol..

[5] Hasiów-Jaroszewska B., Boezen D., Zwart M.P. (2021). Metagenomic Studies of Viruses in Weeds and Wild Plants: A Powerful Approach to Characterise Variable Virus Communities. Viruses.

[6] Hančinský R., Mihálik D., Mrkvová M., Candresse T., Glasa M. (2020). Plant Viruses Infecting Solanaceae Family Members in the Cultivated and Wild Environments: A Review. Plants.

[7] Ma Y., Marais A., Lefebvre M., Theil S., Svanella-Dumas L., Faure C., Candresse T. (2019). Phytovirome analysis of wild plant populations: Comparison of double-stranded RNA and virion-associated nucleic acid metagenomic approaches. J. Virol..

[8] Alvarez-Quinto R.A., Lockhart B.E.L., Olszewski N. (2019). Complete genome sequence of a previously undescribed badnavirus occurring in Polyscias fruticosa L. (*Ming aralia*). Arch. Virol..

[9] Ma Y., Xing F., Che H., Gao S., Lin Y., Li S. (2022). The virome of Piper nigrum: Identification, genomic characterization, prevalence, and transmission of three new viruses of black pepper in China. Plant Dis..

[10] Peng Y., Leung H.C.M., Yiu S.M., Chin F.Y.L. (2012). IDBA-UD: A de novo assembler for single-cell and metagenomic sequencing data with highly uneven depth. Bioinformatics.

[11] Kim D., Langmead B., Salzberg S.L. (2015). HISAT: A fast spliced aligner with low memory requirements. Nat. Methods.

[12] Grabherr M.G., Haas B.J., Yassour M., Levin J.Z., Thompson D.A., Amit I., Adiconis X., Fan L., Raychowdhury R., Zeng Q. (2011). Full-length transcriptome assembly from RNA-Seq data without a reference genome. Nat. Biotechnol..

[13] Altschul S.F., Madden T.L., Schaffer A.A., Zhang J., Zhang Z., Miller W., Lipman D.J. (1997). Gapped BLAST and PSI-BLAST: A new generation of protein database search programs. Nucleic Acids Res..

[14] Sayers E.W., Cavanaugh M., Clark K., Ostell J., Pruitt K.D., Karsch-Mizrachi I. (2020). GenBank. Nucleic Acids Res..

[15] Kumar S., Stecher G., Li M., Knyaz C., Tamura K. (2018). MEGA X: Molecular Evolutionary Genetics Analysis across Computing Platforms. Mol. Biol. Evol..

[16] King A.M.Q., Adams M.J., Carstens E.B., Lefkowitz E.J. (2012). Family—Betaflexiviridae. Virus Taxonomy.

[17] Liu Q., Yang L., Xuan Z., Wu J., Qiu Y., Zhang S., Wu D., Zhou C., Cao M. (2020). Complete nucleotide sequence of loquat virus A, a member of the family Betaflexiviridae with a novel genome organization. Arch. Virol..

[18] Rumbou A., Candresse T., Marais A., Svanella-Dumas L., Landgraf M., von Bargen S., Büttner C. (2020). Unravelling the virome in birch: RNA-Seq reveals a complex of known and novel viruses. PLoS ONE.

[19] Xing F., Hou W., Massart S., Gao D., Li W., Cao M., Zhang Z., Wang H., Li S. (2020). RNA-seq reveals hawthorn tree as a new natural host for apple necrotic mosaic virus, possibly associated with hawthorn mosaic disease. Plant Dis..

[20] Veerakone S., Liefting L.W., Khan S., Pal C., Tang J., Ward L.I. (2021). Partial biological and molecular characterization of a novel citrivirus from *Nandina domestica*. Arch. Virol..

[21] Jia A., Yan C., Yin H., Sun R., Xia F., Gao L., Zhang Y., Li Y. (2021). Small RNA and Transcriptome Sequencing of a Symptomatic Peony Plant Reveals Mixed Infections with Novel Viruses. Plant Dis..

[22] Fiallo-Olivé E., Navas-Castillo J. (2019). Tomato chlorosis virus, an emergent plant virus still expanding its geographical and host ranges. Mol. Plant Pathol..

[23] Fuchs M., Bar-Joseph M., Candresse T., Maree H.J., Martelli G.P., Melzer M.J., Menzel W., Minafra A., Sabanadzovic S., Report Consortium I. (2020). ICTV Virus Taxonomy Profile: Closteroviridae. J. Gen. Virol..

[24] Zhang P., Ma Y., Han T.-T., Smith W.K., Yu J., Cheng Y.-Y., Lu Q.-Y. (2022). First report of a crinivirus infecting mulberry (*Morus alba* L.) in China. J. Plant Pathol..

