# Supplementary material for: Diverse Novel Viruses Coinfecting the Tropical Ornamental Plant Polyscias balfouriana in China
Source: Viruses. 2022 May 24;14(6):1120. doi: 10.3390/v14061120 (PMC9228080; doi:10.3390/v14061120)
Supplement: Supplementary file 1 [file viruses-14-01120-s001.zip › viruses-1698722-SI.pdf]

Table S1. Primers used for the completion of the genomes of PCaV-1 and PCiV-1 and for the detection of the five viruses.

Virus	Primer	Sequences (5'-3')	Coordinates	Direction	Expected size	Usage
**polyscias capillovirus 1 (PCaV-1, genus *Capillovirus*, family *Betaflexiviridae*)**	capillo-F6	TGGAGAAAATGAGTTTGGAAGACGTGCTTC	5615-5644	forward	833 bp	complete genome
	dt-rev*	TGTGTTGGGTGTGTTTGG	3' termini	reverse		
	capillo-F7	GACTCTTCACAGGACCATGTAATTCTAGCT	4161-4632	forward	1498 bp	complete genome & PCR detection
	capillo-R7	AAGCACGTCTTCCAAACTCATTTTCTCCA	5643-5615	reverse		
	capillo-F7.1	CTATGCTGCCAAAGGAATTTGAAGTCA	3719-3745	forward	1145 bp	complete genome
	capillo-R7.1	TATAACTCAGAGGACTTGACAGCATC	4864-4839	reverse		
	capillo-F8	GAATTGATCGAAAAGGTGCCCACAA	2677-2701	forward	1360 bp	complete genome
	capillo-R8	GTATGTAGTATGAGTCTCCACATGC	4036-4012	reverse		
	capillo-F9	GAGAAAGTTAACTAAGAGATTTGATGAAG	951-979	forward	1875 bp	complete genome
	capillo-R9	AAGCCTTGCATCATGTATTTGTGTTCAACTG	2825-2795	reverse		
	capillo-GSP-R1#	CCCGAAAGAGTAAACAAAGTTCAATTCATCAGG	1128-1096	reverse	~1100 bp	complete genome
	LD-oligo(dT) primer	TGTGTTGGGTGTGTTTGGTTTTTTTTTTTTTTT	--	reverse	--	cDNA synthesis
**polyscias citrivirus 1 (PCiV-1, genus *Citrivirus*, family *Betaflexiviridae*)**	citri-F1	CTTAARTGTGGAAAARTGTATTCTGATGC	7216-7244	forward	1091 bp	complete genome
	dt-rev*	TGTGTTGGGTGTGTTTGG	3' termini	reverse		
	citri-F2	CACTCCAACAAAAACTTTGATGATTT	5151-5176	forward	2273 bp	complete genome
	citri-R2	GTGTTGGAGTTTGACCATTTACCATTTTCTT	7423-7393	reverse		
	citri-F3	CATTTGACCATGTCTGTATACTTTTGTC	4123-4150	forward	1387 bp	complete genome & PCR detection
	citri-R3	TTATTCAAAGCACACATATCATCCCCAGC	5509-5461	reverse		
	citri-F4	AATTGCTTTAAGTGGCGAGGAAGACGTTCAG	2490-2520	forward	2002 bp	complete genome
	citri-R4	TGTACAGATGCGTTTGGCAGGTTGGCTCA	4521-4493	reverse		
	citri-F5	GATGTTTTTCCAATTTCTTTGCAGCATATG	989-1023	forward	1831 bp	complete genome
	citri-R5	CATCACAAAGAAAACACCAGGTGTAACACA	2819-2790	reverse		
	citri-F6	CAGTTCTTTTTGTTAGCATTAAGCAGAGT	361-389	forward	1036 bp	complete genome
	citri-R6	GCTTTTTCATCTGTAATTACAACTTCAAATC	1396-1366	reverse		
	Citri-GSP-R2#	ACGCCCAAAATCTTTACCATCTACCATTCTA	476-446	reverse	~500 bp	complete genome
	LD-oligo(dT) primer	TGTGTTGGGTGTGTTTGGTTTTTTTTTTTTTTT	--	reverse	--	cDNA synthesis
**polyscias crinivirus 1 (PCrV-1, genus *Crinivirus*, family *Closteroviridae*)**	clostero_F1	AGCGCGTTTGACGTCTATCGGCTGTTTATAGA	RNA1	forward	~1000 bp	PCR detection of partial RdRp gene
	clostero_R1	TTCGTCTCTGCGCACCTAACTCACCGGCCAA	RNA1	reverse		
**polyscias mosaic virus (PoMV, genus *Badnavirus*, family *Caulimoviridae*)**	poly-bad-F1	CATGATGAGACCAGCAGTAAGTGGCAGC	1160-1187	forward	~500 bp	PCR detection
	poly-bad-R1	TCTGTGCCAAAATGGATTGATCTCCTGT	1671-1644	reverse		
**polyscias rhabdovirus (family *Rhabdoviridae*)**	rhabdo-F1	ATGACAGACGACCTGCTGAGCTTGACAA	--	forward	~500 bp	PCR detection
	rhabdo-R1	AATACTTGATTGTCTCCCATGCCCATGAT	--	reverse		

* This reverse primer 'dt-rev' paired with the gene specific primer were used to amplify the 3' terminal sequence since the complementary DNA (cDNA) was synthesized using LD-oligo(dT) primer.

# This gene specific reverse primer was used in conjunction with the universal primer provided by the SMARTer® RACE 5'/3' kit (Takara Bio) to amplify the 5' terminal sequence of the virus.

Table S2. The amino acid identities of Rep and CP between the PCaV-1 and PCiV-1 and other members in the genus *Capillovirus* and *Citrivirus* of family *Betaflexiviridae*.

**Polyscias capillovirus 1 (PCaV-1)**	**Rep**	**CP**
Apple stem grooving virus	0.66	0.80
Citrus tatter leaf virus	0.65	0.79
Pear black necrotic leaf spot virus	0.64	0.79
Breadfruit capillovirus 1	0.65	0.76
Yacon virus A	0.59	0.54
Avellana capillovirus 1	0.48	0.48
Rubber tree capillovirus 1	0.25	0.34
Currant virus A	0.24	0.31
Mume virus A	0.25	0.31
Cherry virus A	0.24	0.29
**Polyscias citrivirus 1 (PCiV-1)**	**Rep**	**CP**
Citrus leaf blotch virus	0.64	0.72
Dweet mottle virus	0.64	0.72
Nandina citrivirus	0.64	0.66
Paeonia citrivirus A	0.58	0.69
Citrus leaf blotch virus 2	0.55	0.71
Peony yellowing associated citrivirus	0.54	0.70
Citrus leaf blotch virus Ac	0.54	0.70

Table S3. The amino acid identities of HSP70h, RdRp and CP between the PCrV-1 and other formal and unclassified members in the genus *Crinivirus*.

Virus	**HSP70h**	**RdRp**	**CP**	**CPm**	**ORF1a**
Sedum sarmentosum crinivirus	0.78	0.80	0.69	N/A	0.59
Tomato chlorosis virus	0.78	0.79	0.71	0.45	0.58
Bean yellow disorder virus	0.60	0.61	0.33	0.29	0.35
Cucurbit yellow stunting disorder virus	0.60	0.64	0.33	0.27	0.34
Lettuce chlorosis virus	0.60	0.61	0.34	0.27	0.34
Strawberry pallidosis-associated virus	0.59	0.59	0.28	0.21	0.38
Cucurbit chlorotic yellows virus	0.59	0.61	0.34	0.29	0.35
Tetterwort vein chlorosis virus	0.59	0.64	0.34	0.27	0.35
Sweet potato chlorotic stunt virus	0.58	0.68	0.36	0.24	0.40
Diodia vein chlorosis virus	0.57	0.59	0.26	0.21	0.37
Blackberry yellow vein-associated virus	0.57	0.63	0.30	0.21	0.38
Beet pseudoyellows virus	0.57	0.64	0.30	0.21	0.38
Potato yellow vein virus	0.56	0.60	0.33	0.20	0.35
Tomato infectious chlorosis virus	0.50	0.55	0.21	0.14	0.28
Lettuce infectious yellows virus	0.48	0.56	0.27	0.21	0.30

Table S4. The amino acid identities of HSP70h (lower left) and RdRp (upper right) between the formal species in the genus *Crinivirus*.

	BYDV	BPYV	BYVaV	CYSDV	DVCV	LCV	LIYV	PYVV	SPaV	SPCSV	TwVCV	ToCV	TICV
**BYDV**	1.00	0.60	0.63	0.79	0.58	0.86	0.54	0.57	0.59	0.58	0.77	0.62	0.51
**BPYV**	0.64	1.00	0.72	0.62	0.71	0.62	0.58	0.65	0.70	0.64	0.61	0.64	0.57
**BYVaV**	0.64	0.72	1.00	0.64	0.69	0.64	0.57	0.64	0.69	0.63	0.64	0.63	0.56
**CYSDV**	0.74	0.63	0.64	1.00	0.60	0.77	0.54	0.56	0.61	0.61	0.74	0.63	0.51
**DVCV**	0.67	0.69	0.68	0.65	1.00	0.59	0.53	0.63	0.77	0.63	0.61	0.59	0.54
**LCV**	0.84	0.65	0.63	0.73	0.65	1.00	0.54	0.55	0.59	0.58	0.74	0.60	0.51
**LIYV**	0.49	0.49	0.49	0.50	0.51	0.48	1.00	0.53	0.54	0.56	0.53	0.56	0.63
**PYVV**	0.64	0.68	0.68	0.62	0.64	0.64	0.50	1.00	0.61	0.60	0.57	0.61	0.56
**SPaV**	0.66	0.73	0.70	0.63	0.81	0.66	0.51	0.65	1.00	0.63	0.61	0.59	0.54
**SPCSV**	0.64	0.64	0.64	0.62	0.64	0.63	0.51	0.62	0.64	1.00	0.62	0.68	0.54
**TwVCV**	0.78	0.63	0.64	0.71	0.67	0.79	0.51	0.62	0.65	0.66	1.00	0.62	0.52
**ToCV**	0.61	0.60	0.59	0.60	0.59	0.60	0.51	0.58	0.61	0.61	0.60	1.00	0.56
**TICV**	0.55	0.53	0.53	0.53	0.52	0.53	0.64	0.54	0.53	0.53	0.53	0.53	1.00

The values of the amino acid identities greater than 0.75 were in red. The full names of the criniviruses are listed below: bean yellow disorder virus (BYDV), beet pseudoyellows virus (BPYV), blackberry yellow vein-associated virus (BYVaV), cucurbit yellow stunting disorder virus (CYSDV), diodia vein chlorosis virus (DVCV), lettuce chlorosis virus (LCV), lettuce infectious yellows virus (LIYV), potato yellow vein virus (PYVV), strawberry pallidosis-associated virus (SPaV), sweet potato chlorotic stunt virus (SPCSV), tetterwort vein chlorosis virus (TwVCV), tomato chlorosis virus (ToCV), tomato infectious chlorosis virus (TICV).

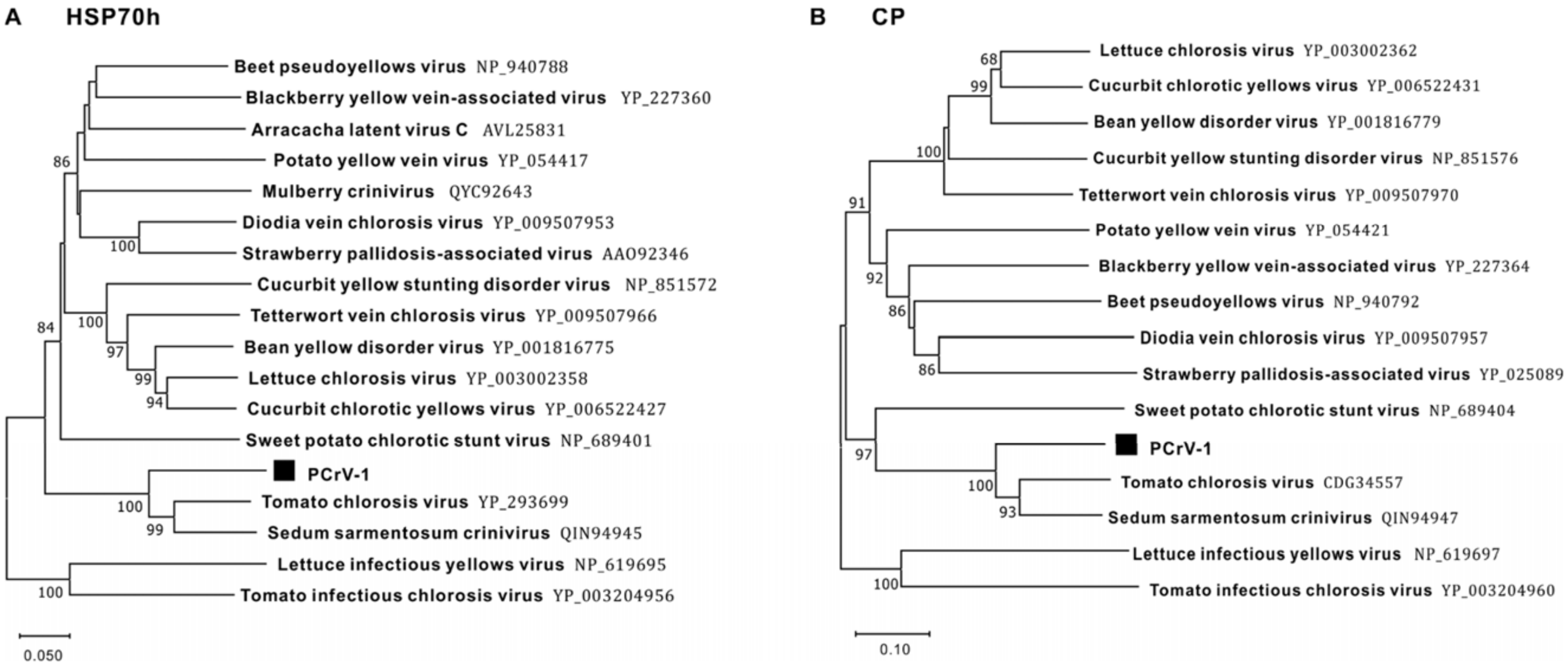

Figure S1. Phylogenetic trees constructed based on the HSP70h and CP amino acid sequences of formal and tentative members in the genus *Crinivirus*.

The trees was constructed using the neighbor-joining method, and the statistical significance of branches was evaluated by bootstrap analysis (1000 replicates). Bootstrap values above 50% are shown. The new virus polyscias crinivirus 1 (PCrV-1) is indicated with black square.

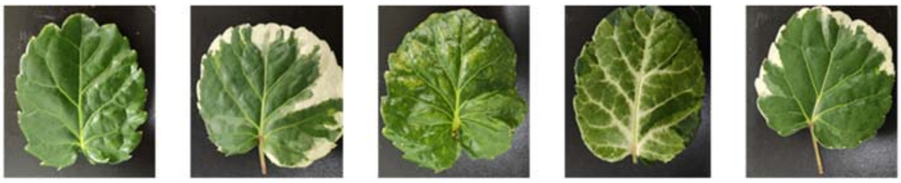

	1	2	3	4	5
PCaV-1	+	+	-	+	+
PCiV-1	+	+	-	+	-
PCrV-1	+	+	-	+	+
PoMV	+	+	-	+	+
Rhabdovirus	-	+	-	-	+

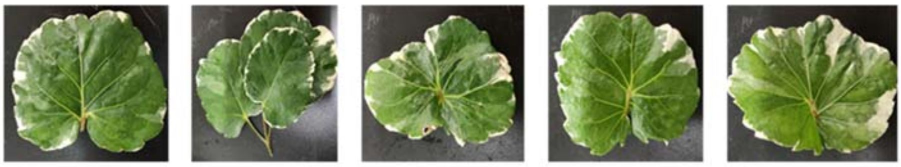

	6	7	8	9	10
PCaV-1	-	+	+	+	+
PCiV-1	-	+	+	+	+
PCrV-1	+	+	+	+	+
PoMV	+	+	+	+	+
Rhabdovirus	+	-	-	-	-

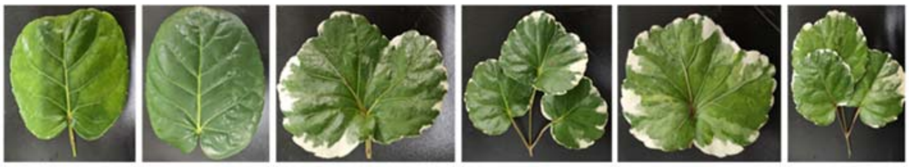

	11	12	13	14	15	16
PCaV-1	+	-	-	+	-	+
PCiV-1	+	+	-	+	-	+
PCrV-1	+	+	-	-	-	+
PoMV	+	+	-	+	-	+
Rhabdovirus	-	+	+	+	-	-

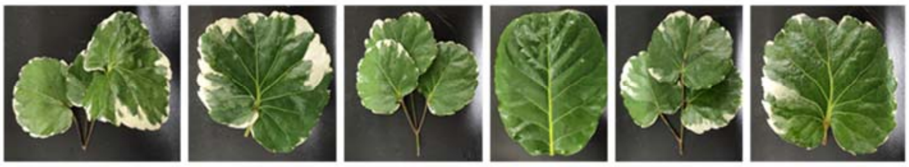

	17	18	19	20	21	22
PCaV-1	+	+	+	+	+	-
PCiV-1	+	+	+	+	+	-
PCrV-1	+	+	+	+	+	+
PoMV	+	+	+	+	+	+
Rhabdovirus	-	-	-	+	-	-

Figure S2. Leaf morphology of 22 *Polyscias balfouriana* samples collected from Xinglong Tropical Botanical Garden, Hainan, China in 2021 and the occurrence of the new viruses (PCaV-1, PCiV-1, PCrV-1, PoMV, and the rhabdovirus) in these samples. Of the 22 samples, 17 had rounded leaves edged in white (*P. balfouriana* 'Marginata'), three *Polyscias balfouriana* samples (ID. 11, 12, 20) had reniform thick leaves two to three times larger in size than those of P. balfouriana 'Marginata', one sample (ID. 3) had yellow spotted leaves, and one sample (*P. balfouriana* 'pennockii', ID. 4) had chlorotic and vein banding leaves. The target viruses are polyscias capillovirus 1 (PCaV-1), polyscias citrivirus 1 (PCiV-1) and polyscias crinivirus 1 (PCrV-1), polyscias mosaic virus (PoMV) and the potential novel rhabdovirus. The IDs of the samples are indicated below the photos. The positive and negative results are indicated with "+" and "-" respectively and samples with no detected viruses (no. 3, 15) are in grey background in the table.
